# Bibliographical Notices

**Published:** 1855-11

**Authors:** 


### BIBLIOGRAPHICAL NOTICES.

*Elements of Medicine: a compendious view of Pathology and Therapeutics; or, the History and Treatment of Diseases.* By Samuel Henry Dickson, M. D., L. L. D., Professor of the Institutes and Practice of Physic in the Medical College of the State of South Carolina. Philadelphia, Blanchard & Lea.

It is a duty, in our opinion, incumbent upon every one who occupies for any length of time, the responsible position of Teacher of Medicine, to give forth occasionally the fruits of his accumulated study and experience to the world. However thorough may be his acquirements or extensive his information, so long as they are imparted only to a few students, he cannot but be regarded as an unprofitable servant to the profession at large. This natural demand upon those whose superior talents and position have given them advantages denied to the many, although a just claim, is, however, too often disregarded, particularly in our own country; and it is with sincere gratification, therefore, that we notice the appearance of Prof. Dickson's work upon the Elements of Medicine.

By far the largest portion of the work is devoted to Special Pathology and Therapeutics. As an introductory to his exposition of those branches, the author very properly premises a view of General Pathology, a department of medical science too little and too loosely studied by the great mass of the profession. The great advantages which a practitioner secures from a general and comprehensive view of disease, which can only be obtained from a proper appreciation of the nature, causes, symptoms, progress and pathological results, which are the common property of all diseases, cannot well be over estimated. Without such general principles to guide him at the bedside, he can hardly fail, however well taught in other respects, to degenerate into a mere routinist, with his particular remedy for every disease, no matter what may be its type or complication or natural duration. A philosophical physician, competent to every emergency, it is impossible he can ever be.

The first part of the work, which, as we have just said, is on General Pathology, is devoted to the consideration of the nature of disease, etiology, the seats of disease, the phenomena of disease and the tendency of diseases. Each of these subjects is briefly, and, in general, judiciously sketched. Under the head of "Incidental Causes of Diseases," we notice the following remarks upon the influence of the corset or stay in the production of disease: "The female corset or "stays," with their appendages, have been subjects of reiterated and severe animadversion. They are susceptible, however, I think, of a fair defence. They are not necessarily hurtful, but do injury only by excess or want of adaptation. They give a graceful support to the form, which, in a large majority of instances, they unequivocally improve by imparting an air of firmness and neatness. The athlete always girds his loins for the contest, whether of wrestling or running; and even the charming mother of Cupid became more irresistible when she encircled her symmetrical waist with the cestus. Matrons feel the need of 'Supporters,' and encourage the ingenuity of those who provide them with suitable arrangements.

The evils of the corset have been, I am persuaded, greatly exaggerated. If laced too tightly, it must compress the thorax and impede the respiration; it may also constrain the viscera, and render the motion of the body inconvenient and awkward. Yet I do not remember, in forty years' practice, to have met with any definite example of deformity or injury from this cause. I have more than once seen the breasts hurt and inflamed by having the steel-piece or whalebone in front too broad, so as to press upon them, but this was soon relieved."

The influence of the corset in producing *consumption* is very fully and philosophically described by Dr. Walshe, in the Appendix to his treatise on the Diseases of the Lungs and Heart, 2d edition, to which we refer those of our readers who are desirous of more information upon this subject. His concluding remarks, in which, it will be seen, he differs somewhat from our author, are these: "If the abuse of stays produces consumption, its power to do so most indubitably remains to be proved; and while the laws of an enlightened pathology point to the excessive improbability of an essentially diathetic disease springing from a mechanical cause, I entreat you not to adopt the popular creed, that 'stays cause consumption,' unless on direct and unimpeachable logical evidence. There is quite enough in the demonstrable evils entailed by tight lacing to justify you in warring against the abuse; you have no need to support your arguments by the unfair appeal to an imaginary mischief."

Our author's affirmative belief in the contagious nature of certain diseases, yellow fever, cholera, &c., is well known to the profession by his previous writings. His views may be summed up as follows: Two elements, he says, are "essential to the description of contagion as a cause of disease; first, that it should be *germinal*, that is, self-multiplying, reproductive; and, second, that this reproduction should depend upon, or be favored by, the very *processes of disease* which itself gives rise to." It may, however, originate spontaneously or independently, and as nothing inorganic can propagate itself, it must be organic in its nature. The organisms, in which this power of reproduction resides, and which have never yet been insulated or discovered, must possess an affinity for the *human* constitution. As a known contagious disease, variola for example, may reproduce itself by the insertion of matter into a wound from a vesicle, and also act efficiently *at a distance*, we must in the latter instance ascribe the phenomena either to the radiation or diffusion of the same *materies morbi* which is contained in the scab; or "we must assume the production and elimination of two forms of efficient contagious matter. But the first is already shown to be ultra-microscopic in the minuteness of the atoms in which it consists, and, therefore, I see no difficulty in its elimination and radiation from the deseased body." Dr. Dickson is of opinion that analogy is a very unsafe guide in this question. Because a palpable contagious matter is evident in some diseases, whilst others show, *in addition*, an impalpable or diffusible contagion, effective at a distance, physicians, he thinks, are too apt to believe that a third class exhibiting the property of diffusiveness only, and which have never yet been conveyed from a diseased to a healthy body *directly*, such as typhus, pertussis, puerperal fever, yellow fever and cholera asphyxia, cannot, therefore, be contagious. A capacity for self-multiplication, however, exists in each of these; each of them, too, proceeds according to its own laws of appearance, increase and decline; "but in all, the phenomena of their extension and propagation; their great diversity of localities, their attendance upon migratory masses; their prompt multiplication under fostering contingencies connect them closely with the undoubted, undenied contagions." If the organic, self productive character of this class of diseases be denied, insuperable objections, according to our author, present themselves. Taking cholera for an example, great masses of its poison must exist on the earth's surface. This poison must either be generated by local causes everywhere, where the disease is present, or it must originally have been produced in great abundance to have diffused itself so widely. Both of these opinions are, our author thinks, indefensible. He believes also, that all contagions are contingent. Yellow fever, dysentery, and the plague, are contingent upon seasons, climate, and temperature, while small pox, measles, scarlet fever, and cholera, though they defy all *known* contingencies, have their individual preferences and exemptions.

To determine the contagious character of a disease, he says:

"Inoculation will decide where any palpable matter is found. Where there is no visible product, we must be guided by rational inference from obscure facts, such as:

1. Repeated spread among those surrounding a sick man.
2. The occurrence of repeated cases upon exposure to various fomites; these are circumstances which give obvious reasons for the belief of the contagiousness of any maladies of which they are predicable.
3. Progressive extension from a first observed locality.
4. A decided preference for dense populations.
5. Repeated migration with travelling persons or bodies.
6. A preference for the ordinary thoroughafares by sea and land."

Our limits permit us only to observe here that these tests for the determination of the contagious nature of diseases, are valueless in our opinion, as regards one of the diseases in question. How does the first rule which carries in the opinion of the majority of persons the greatest weight with it, apply to yellow fever? During the prevalence of that disease in Philadelphia in 1853, it was confined to a certain locality in the vicinity of the river. Although numerous patients affected were carried to the Pennsylvania Hospital, which is distant half a mile or more from the infected neighborhood, and were there placed indiscriminately with other cases in the wards, and though several autopsies were made, not a single instance occurred of propagation of the disease. The same immunity was observed in places to which persons fled, with the seeds of disease in them, from Norfolk and Portsmouth. We might except to some of the other rules also. We have not space, however, for the discussion of the subject here. That the profession is not more fully agreed upon such an important question, involving as it does, the most serious consequences, is much to be regretted.

Under the head of Special Pathology and Therapeutics, are included the Classification of Diseases,—Diseases of the circulatory system, of the digestive system, of the respiratory system, of the sensorial system, of the motory system, and of the excernent system. We regret that we have no space to copy several passages that we had marked for that purpose, and upon which we desired to make some comments.

One of the chief merits of Dr. Dickson's work is the excellence of its style. Always smooth, clear, and flowing, it is at times characterized by a warmth and glow of language, not often found in didactic treatises. The reader will find in the following passage an example of the finished and ornate mode of expression with which the author frequently embellishes the most ordinary topic:

"Of all occupations, it may readily be conceded, that agricultural pursuits are most friendly to physical well-being. The labors of the farmer, though regular, are sufficiently diversified, while they are free from the listlessness which is apt to follow desultory exertions; though active and vigorous, they are not so urgent as to fatigue necessarily or exhaust the strength. Nor do they involve generally any very unpleasant or injurious exposure. When the fields are covered with snow, and the streams are ice-bound; when the soil is hardened by frost, when the cold winds whistle and the wintry storm blows loud; he shelters himself within doors engaged in such labor as may be done under a roof, or suspends all toil for a time. When spring unbinds the fountain and thaws the frost and snow, and loosens the solid earth, he breaks up the clods with his plough and harrow, and sows and plants with cheerfulness and hope. During the heats of summer, he may divide his time so as to avoid the noontide glare of the sun, and pass the sultriest hour in the shade; the mornings and evenings of the lengthened day being sufficient for his out door work. The declining and mellow autumn calls him again into the field to gather the fruits of his labor in the glad harvest home, after which he again prepares and enriches the soil. Such is the charming picture of agricultural life given us by the poets and rustic writers of England, and applicable as well to the eastern, middle, and north-western States of this continent. Far different, however, is the planter's life of our south and south-western territory."

In concluding this too short and incomplete notice of Dr. Dickson's Elements, in which our principal object has been to exhibit the author's reasons for his belief in the contagious nature of yellow fever, cholera, &c., we would take occasion to recommend it to the profession as a work containing much varied information conveyed in a very agreeable style.

---

*A Practical Treatise on the Diseases of the Eye.* By WILLIAM MACKENZIE, M. D., &c., *to which is prefixed an Anatomical Introduction, Explanatory of a Horizontal Section of the Human Eye-ball.* By THOMAS WHARTON JONES, F. R. S., &c., *with one hundred and seventy-five illustrations. From the fourth revised and enlarged London Edition. With notes and additions*, by ADDINELL HEWSON, A. M., M. D., &c. Philadelphia: Blanchard & Lea. 1855.

If the number of editions through which a work has passed be any criterion of its merits, and there can be little doubt that, in most instances, it is a very safe one, Dr. Mackenzie's Treatise on the Diseases of the Eye is entitled to very strong claims upon our attention. It has passed through three large editions in England and is now in its fourth; and has been twice re-produced in our own country, besides having been "translated and published in the three best known languages of modern Europe, German, French, and Italian." So general and marked a testimony to the estimation in which it is held throughout the whole civilized world, ought to be sufficient, we think, to satisfy any one previously ignorant of its qualifications of its trustworthy character.

A large amount of new matter, the author states, has been added by him, to bring the work up to the level of the present state of the science. Additional wood cuts have also been inserted, and, under each head, he has introduced the most remarkable synonymes, with special references to those works where the most truthful pictures of the disease may be found.

The chief peculiarity of Dr. Mackenzie's Treatise consists in its numerous references to other writers, and in the copious supply of its cases. In these respects it has no equal in the English language. By the student desirous to obtain a *thorough* acquaintance with any one subject, and by the busy practitioner, anxious for more detailed instruction than is usually to be found in systematic treatises, these aids will be estimated at their proper value. Who is there, indeed, that has not felt the want of quick and accessible information on such matters ?

Its other and rare merits, the sound and excellent principles it inculcates, and the good judgment shown throughout, make it a safe and efficient guide for the student.

The Editor informs us in his preface that—

"Numerous wood-cuts have been inserted, and such additions have been made as, it is hoped, will prove acceptable to the American reader. They relate chiefly to matters of a practical character, and are embraced in brackets, with the initial H. appended. Amongst these will be found a short account of the ophthalmoscope, and the various conditions which have thus far been revealed by its use, and to which the author has scarcely alluded."

The Editor's additions are generally brief. They are upon the subcutaneous division of the periosteum; the use of red oil for the prevention of a black eye; the advantages of the "Donna Maria gauze" as a dressing for wounds of the eye-lids: a description of Mr. Wilde's ring-forceps; of Mr. Himly's Entropion forceps; M. Desmarres' method of everting the upper-lid; the use of a series of probes (Dr. Hays' method) for obstruction of the puncta; the employment of tubes of ivory, (Dr. Pancoast's plan), for styles; Dr. Macdonald's method of determining slight strabismus; a case of the removal of a piece of iron from the cornea; arguments for disbelief in the metastasis of gonorrhœa; scrofulous abscesses of iris, with a case; pathological changes revealed by the ophthalmoscope in the choroid, vitreous body, &c.; the proper mode of using blue-stone; Dr. Pancoast's method for displacing hard cataract; Dr. Hays' improvement of the cataract needle; Jaeger's keratome, &c. In his account of the ophthalmoscope he very properly guards his reader against expecting too much from the use of that instrument, considering its employment, unless in those cases where disease is confirmed and incurable, as both injurious to the eye, and liable, from its producing an excited condition of the structures examined, to lead the observer astray. "In these incurable cases, though," will be found the great value of the instrument; for it, and it alone, will often enable us to set aside, as Mr. Dixon justly observes, "as positively hopeless, a large number of cases formerly termed '*amaurotic*' or '*nervous*,' which were assumed to be curable, because their real nature could not be demonstrated."

In a late excellent review of Mr. Dixon's work in the Edinburg Medical Journal, the writer, speaking of this instrument says, "To a hopelessly disorganized eye, little can be done; here it may be curious to see what changes have taken place in the retina and vitreous body; but when there is still a possibility of vision being saved or improved, we hold that it is injudicious to irritate the retina, by concentrating more light upon it." Then quoting Mr. Dixon's language as above given, he adds, "we much question if it will be found of any real practical utility." In which opinion we most cordially agree.

The editor states in his second note, that the whole proper substance of the cornea "consists of a mixture of yellow and white fibrous tissue." With our understanding of the physiological and anatomical properties of yellow fibrous tissue, we cannot comprehend how this can be. Its tissue is peculiar according to Kolliker, consisting "of a fibrous substance, closely allied to connective tissue, but which, according to J. Müller, affords, when boiled, not *gelatin* but *chondrin.*"

For our own part, we are free to confess, that we consider the addition of notes to such a work as Mr. Mackenzie's mere supererogation. As a general rule, we prefer, and we think the profession prefers, to read a reprint as it comes from the writer, without any additions. They interrupt the continuity of the narrative, are often trifling in their nature, or such as would naturally suggest themselves to the mind of the reader, and occasionally, though we do not wish to be understood now as referring to the work before us, they intrude opinions entirely at variance with those of the author. We except, from this censure, such works as have been essentially altered in their character by the authorised emendations of the editor, or where the original is out of print, or the author dead, especially where the editorship is in the hands of those whose experience eminently fits them for the task.

The fact is not to be denied that the manner in which foreign works are usually edited in this country, is anything but creditable to those who undertake that office. Without now questioning the propriety of their assumption of duties unsanctioned by the author, they too seldom give us the results of American science, or much less, of extensive reading. To correct the proof sheets, and add a few notes, seem to be considered all that is necessary. We could mention works where the additions have not amounted to more than a page or two, and yet the editors' names are in the title page, and the books are partly called after them. The same notes, also, (we know them by heart,) but slightly altered, are repeated in certain works *ad nauseam.* It is very desirable that all this should be changed, and that these gentlemen should be held to a much stricter accountability.

The typography and illustration of the book are excellent.

---

*A Manual of Clinical Medicine and Physical Diagnosis.* By T. H. Tanner, M. D., &c., Physician to the Hospital for Women, &c. To which is added the Code of Ethics of the American Medical Association. Philadelphia, Blanchard & Lea, 1855.

This compact little volume is admirably calculated to guide and direct the student in studying disease at the bed-side. It embraces in its several chapters, the clinical study of disease; the instruments employed in the diagnosis of disease; disease: the various circumstances which modify disease; the symptoms and signs of disease; the physical diagnosis of disease; general observation of the diagnosis of thoracic diseases: the diagnosis of diseases of the skin: parasitic worms found in the human body: and the chemical and microscopical examination of the blood, expectoration, vomited matters and urine.

It is simply and clearly written, briefly, and to the point, and contains in a small compass an uncommon amount of useful information.

We trust that it will meet with the success it so richly deserves.

---

*The Physician's Visiting List, Diary, and Book of Engagements for* 1856. Philadelphia, Lindsay & Blakiston, 25 South Sixth St. above Chestnut.

We believe that no physician who has once used the "Visiting List," would ever afterwards like to be without it. To those who have never employed it we can recommend it, from personal experience. It contains an Almanac, a table of poisons and their antidotes, a table for calculating the period of utero-gestation and blank leaves for visits, memoranda, engagements, &c.

